# General anesthesia with remimazolam for emergency cesarean section in a patient with acute infective endocarditis: a case report

**DOI:** 10.1186/s40981-023-00645-5

**Published:** 2023-08-24

**Authors:** Shizuka Yamamoto, Yoshimasa Oyama, Mika Sasaki, Mayu Miyagoshi, Shigekiyo Matsumoto, Takaaki Kitano

**Affiliations:** Department of Anesthesiology and Intensive Care Medicine, Faculty of Medicine, Oita University, 1-1 Idaigaoka-Hasamamachi, Yufu City, Oita 879-5593 Japan

**Keywords:** Remimazolam, Cesarean section, General anesthesia, Acute heart failure, Infective endocarditis

## Abstract

**Background:**

The anesthetic management of pregnant women with acute heart failure remains challenging with regard to maintaining the hemodynamic status of the mother and baby. The likelihood of decreased blood pressure is lower with remimazolam than with propofol. However, there is no report of general anesthesia with remimazolam for cesarean section.

**Case presentation:**

The patient was a 34-year-old pregnant woman who was diagnosed with acute heart failure associated with infective endocarditis. We performed cesarean section under general anesthesia using remimazolam, with percutaneous cardiopulmonary support on standby. The mother’s mean blood pressure was maintained above 65 mmHg during the surgery, without catecholamines or vasopressors. The infant’s Apgar scores were 4 at 1 min and 7 at 5 min.

**Conclusion:**

Cesarean section was successfully performed under general anesthesia with remimazolam in a pregnant patient with acute heart failure. Further studies are needed to clarify the association between remimazolam and neonatal hypotension.

## Background

The anesthetic management of pregnant women with acute heart failure remains challenging with regard to maintaining the hemodynamic status of the mother and baby. Generally, propofol is used for general anesthesia in cesarean section, but it may cause hemodynamic collapse during anesthesia. The likelihood of decreased blood pressure during anesthesia is lower with remimazolam than with propofol, but there is no report of general anesthesia with remimazolam for cesarean section [1]. Here, we report a case of a pregnant patient with acute heart failure who underwent general anesthesia with remimazolam for emergency cesarean section.

## Case presentation

**Fig. 1:**
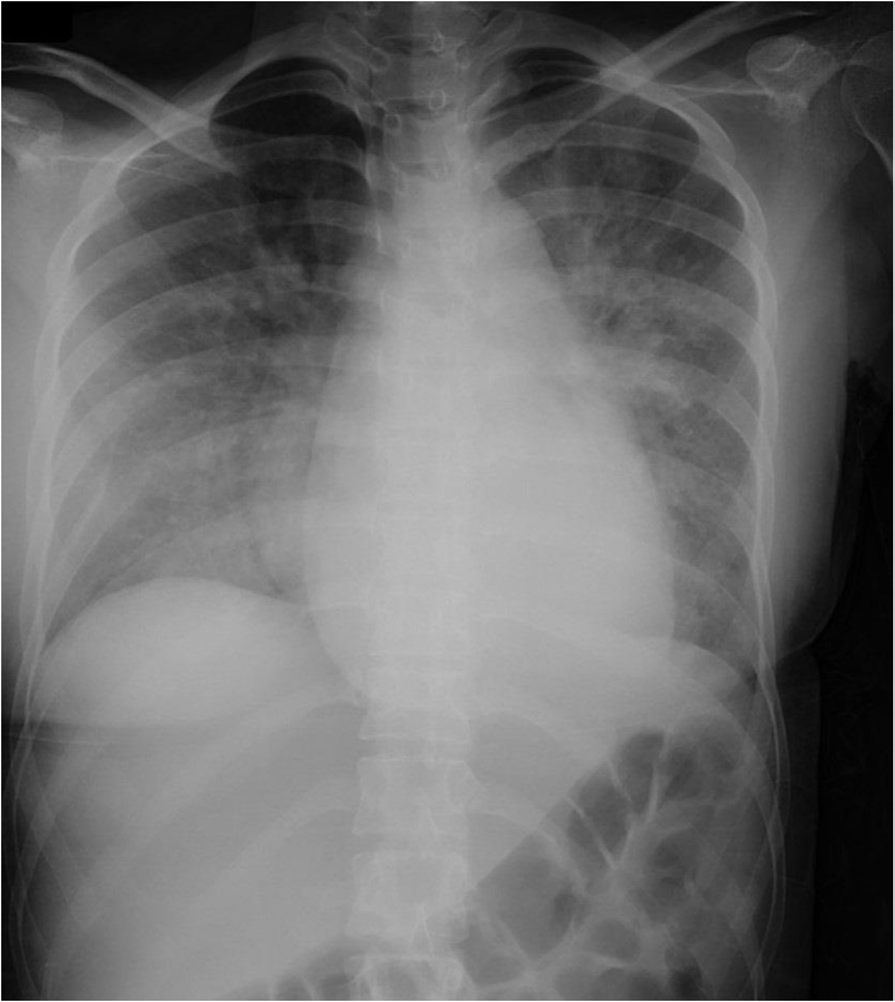
Chest X-ray on the day of cesarean section. The image demonstrates the enhancement of pulmonary vessels

The patient was a 34-year-old pregnant woman (height, 156 cm; body weight, 49 kg). She had no relevant history of comorbidities or medications, and her pregnancy course was unremarkable. At 24 weeks and 4 days of gestation, she experienced epigastric pain and was admitted to another hospital. *Streptococcus mitis*/*oralis* was detected from two sets of blood cultures, and antibiotic therapy was initiated. At 25 weeks and 1 day of gestation, she experienced shortness of breath. Chest X-ray demonstrated features of acute pulmonary edema (Fig. 1). An echocardiogram confirmed the diagnosis of severe mitral regurgitation from infective endocarditis. She experienced acute heart failure (New York Heart Association [NYHA] class IV) and was then transferred to our hospital at 25 weeks and 2 days of gestation.

We planned to perform emergency cesarean section on the same day but before cardiac surgery, as cardiac surgery during pregnancy is associated with a high fetal mortality rate [2]. The obstetrician performed the abdominal ultrasonography examination and confirmed that the fetal heart rate and amniotic fluid level were normal. Although we considered spinal anesthesia, the heart failure symptoms of the patient were too severe to maintain supine position. Her last meal and drink were 10 h prior surgery. She did not have any risk of difficult airway except for pregnancy. Finally, we selected general anesthesia with percutaneous cardiopulmonary support (PCPS) on standby. Before anesthesia induction, we placed an arterial catheter in the left radial artery. The transducer was connected to the radial arterial line to evaluate the arterial-based cardiac index (FloTrac™, Edwards Lifesciences). Thereafter, we inserted a central venous catheter via the right internal jugular vein. A 4-Fr sheath was placed into the right internal jugular vein and the right femoral vein to establish vascular access for PCPS.

We confirmed the fetal heart rate to be 160 beats per minute, which showed reassuring fetus status, then initiated continuous administration of remifentanil (0.03 μg/kg/min) and remimazolam (6 mg/kg/h). With the patient under a fasting condition, we selected continuous administration of remimazolam (6 mg/kg/h) until confirming that she had fallen asleep, instead of rapid sequence induction. She fell asleep after 90 s of 7.2 mg remimazolam administration, which was eventually reduced to 1.2 mg/kg/h. Her spontaneous breathing was maintained until just before she fell asleep. We subsequently placed a tracheal tube 1 min after administration of 50 mg of rocuronium, and the cesarean section was immediately started.

**Fig. 2:**
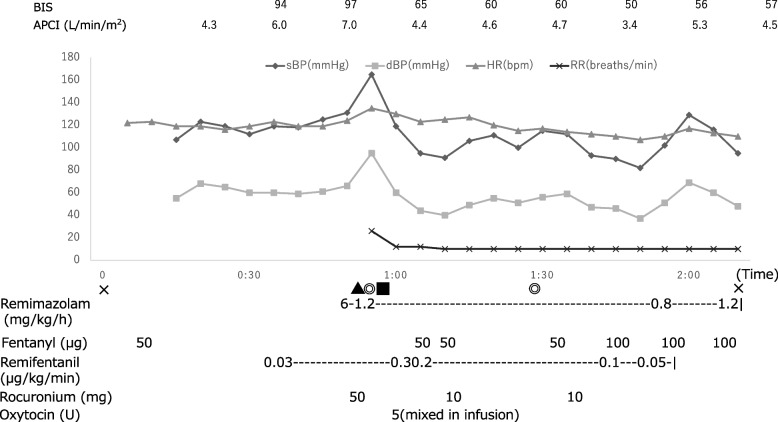
Anesthesia record during emergency cesarean section. BIS, bispectral index; APCI, arterial pressure-based cardiac index; sBP, systolic blood pressure; dBP, diastolic blood pressure; HR, heart rate; RR, respiratory rate; × , start of anesthesia or end of anesthesia; ▲, tracheal intubation; ■, baby birth; ◎, start of surgery or end of surgery

She delivered a male baby weighing 926 g after 4 min of the operation. Total dosage of remimazolam until the delivery was 12.1 mg. The infant’s Apgar scores were 4 at 1 min and 7 at 5 min, and the pH of the umbilical artery was 7.35. As the baby did not cry, the neonatologist promptly intubated and administered surfactant. The mother’s mean blood pressure was maintained above 65 mmHg during the surgery, without catecholamines or vasopressors. The bispectral index values were around 50 to 65. After the cesarean section, her hemodynamic condition was stable, and she was transferred to the intensive care unit (ICU) under intubation (Fig. 2).

She underwent mitral annuloplasty on postoperative day 2, and she was weaned from vasopressors and ventilatory support. She was discharged to the ward from the ICU on day 5 after the cesarean section. The infant was diagnosed with patent ductus arteriosus and underwent ductus arteriosus ligation at 14 days old. He was weaned from ventilatory support at 35 days old and was discharged at 96 days old.

We have obtained the patient’s written consent for the publication of this case report.

## Discussion

To our knowledge, this is possibly the first case report of a patient with acute heart failure who underwent general anesthesia with remimazolam for emergency cesarean section.

In this case, the patient’s mean blood pressure was maintained above 65 mmHg without any vasopressor. A previous study suggested that the likelihood of hypotension is lower with remimazolam than with propofol in patients with heart failure of NYHA class II or III [3]. A recent study demonstrated that remimazolam could be safely used as an induction agent in patients with severe aortic stenosis [4]. Thus, we consider that remimazolam is useful in patients with an unstable hemodynamic status. Hypotension can cause problems for both the mother and fetus. Infants of mothers with hypotension have been reported to exhibit substantial acidosis [5], and maternal hypotension has been shown to be associated with abnormal neurobehavioral activities [6]. Thus, it is desirable to avoid long-term hypotension during cesarean section.

There is a lack of clinical data on the placental transfer of remimazolam. Remimazolam is a midazolam derivative added with a carboxylic ester moiety. In a study of anesthesia for cesarean section, general anesthesia was induced with a high dose of midazolam (0.3 mg/kg) and placental transfer of midazolam was detected. Moreover, the umbilical/maternal concentration ratio was 0.66 immediately after the umbilical cord was clamped [7]. In a randomized trial, administration of 0.2 mg/kg of midazolam with rapid sequence anesthetic induction resulted in lower Apgar scores when compared with thiopental [8]. Since the lipid/water solubility, molecular weight, and protein binding features of remimazolam are similar to those of midazolam, remimazolam may transfer across the placenta from the mother to the fetus. Remimazolam is rapidly metabolized into inactive metabolites by carboxylesterase 1 (CES 1) in liver tissues, and its duration of action is short. Drug metabolism enzymes present in the liver are functionally immature in fetuses, and their activity begins to increase after birth. CES 1 expression is developmentally regulated from infancy to adulthood, and the expression level in neonates is 7% of that in adults [9]. Taken together, care should be taken regarding sedation and respiratory depression in infants after delivery. A further study is required to determine the placental transfer of remimazolam and the pharmacokinetics of remimazolam in fetuses.

In cases of extremely preterm delivery, neonatal ventilatory support may be inevitable regardless of the method of anesthesia. A cohort study showed that a pediatrician is required in cases of emergency cesarean section that is performed under general anesthesia, and in the presence of fetal distress [10]. In this case, we communicated with the neonatologist before surgery regarding the possibility of neonatal respiratory depression. Thus, we could focus on maintaining the hemodynamic status of the mother. The ultimate goal was to save the lives of mother and child. Moreover, in cases with undesirable neonatal respiratory depression, the effects of remimazolam can be antagonized by flumazenil.

In summary, total intravenous general anesthesia was successfully achieved with remimazolam for emergency cesarean section in a pregnant patient with acute heart failure. We successfully managed the mother’s hemodynamic status during the surgery, but the baby had neonatal respiratory depression and needed intubation. Although the gold standard anesthesia method for pregnant with acute heart failure is still unclear, remimazolam administration may be considered. Further studies are needed to assess the usefulness of remimazolam for cesarean section.

## Data Availability

The datasets used and/or analyzed during the current study are available from the corresponding author on reasonable request.

## Abbreviations

CES 1: Carboxylesterase 1
ICU: Intensive care unit
NYHA: New York Heart Association
PCPS: Percutaneous cardiopulmonary support
